# The role of miRNAs in the development of brain metastases originating from lung adenocarcinoma

**DOI:** 10.3389/fgene.2026.1769972

**Published:** 2026-02-20

**Authors:** Bernadett Torner, Álmos Klekner, István Balogh, András Penyige, Dóra Géczi, Tekla Gáspár, Gréta Geszti, Zsuzsanna Birkó

**Affiliations:** 1 Department of Medical Genetics, Faculty of Medicine, University of Debrecen, Debrecen, Hungary; 2 Department of Neurosurgery, Faculty of Medicine, University of Debrecen, Debrecen, Hungary

**Keywords:** biomarker panel, brain tissue, invasion, lung adenocarcinoma brain metastasis, miRNAs, next-generation sequencing

## Abstract

**Introduction:**

Brain metastases (BMs) represent most malignant lesions of the central nervous system. Lung cancer—particularly lung adenocarcinoma (LUAD, ∼25%)—is the most common source of BMs. MicroRNAs (miRNAs) play a crucial role in regulating gene expression, thereby contributing to tumor progression and metastatic spread. Identifying these regulatory molecules may enable a deeper understanding of the mechanisms driving LUAD brain metastasis (LUAD-BM) development and reveal therapeutic targets to prevent or limit disease progression.

**Methods:**

Next-generation RNA sequencing (RNA-seq) was performed on six LUAD-BM and six non-tumorous human brain tissue samples to assess miRNA expression profiles. Additionally, RNA-seq data from 20 primary LUAD and 15 normal lung tissue samples were obtained from The Cancer Genome Atlas (TCGA) database. MiRNAs showing the most pronounced alterations in LUAD-BM samples were selected for validation by real time quantitative polymerase chain reaction (RT-qPCR).

**Results:**

Analysis of RNA-seq data identified 229 differentially expressed (DE) miRNAs between LUAD-BM and control samples. Functional annotation analysis indicated that these DE miRNAs are key regulators of tumorigenesis and metastasis. Using the Mann–Whitney U test, ten miRNAs were confirmed to differ significantly between LUAD-BM and normal brain tissue. Receiver operating characteristic (ROC) curve analysis demonstrated their diagnostic potential. Among the ten validated miRNAs, miR-200c-3p, miR-146b-5p, and miR-3934-5p showed distinct expression patterns between primary LUAD and LUAD-BM, while miR-10a-5p, miR-210-3p, and miR-130b-3p exhibited stepwise dysregulation along the normal lung–LUAD–LUAD-BM axis, suggesting their involvement in metastatic progression.

**Conclusion:**

We identified ten miRNAs that showed preliminary ability to differentiate LUAD-BM from normal brain tissue. These findings indicate possible diagnostic and therapeutic implications. Among these, six miRNAs showed significant expression changes along the normal control–primary LUAD–LUAD-BM axis, highlighting their potential as biomarkers and therapeutic targets in BM development.

## 1 Introduction

Non-small cell lung cancer (NSCLC) accounts for approximately 85% of all lung tumor cases and is considered one of the leading causes of cancer-related mortality world-wide (Molina et al., 2008). Lung adenocarcinoma (LUAD) represents the most common histological sub-type of NSCLC, around 40%–50% of all lung cancer patients are diagnosed with it (Herbst et al., 2018). In addition, LUAD frequently gives rise to distant metastases, particularly in the brain. Approximately 25% of patients with LUAD develop brain metastases (BMs) during the course of their disease (Lukas et al., 2014). LUAD brain metastases (LUAD-BM) are often associated with severe neurological symptoms and poor prognosis, with a median overall survival of 4–15 months (Sperduto et al., 2020).

From a clinical perspective, LUAD-BMs are often diagnosed synchronously with the primary tumor. Treatment strategies include a multimodal approach combining local therapies—such as stereotactic radiosurgery or whole-brain radiotherapy–with systemic treatments, including tyrosine kinase inhibitors in case of epidermal growth factor receptor (EGFR) mutant NSCLC patients (Le Rhun et al., 2021).

The molecular mechanisms underlying brain-specific metastasis in LUAD remain incompletely understood. However, current evidence suggests that they involve a complex interplay between tumor-intrinsic factors, such as gene expression signatures and microRNA (miRNA) profiles (Najjary et al., 2023). Certain oncogenic drivers, particularly EGFR mutations and anaplastic lymphoma kinase rearrangements, have been associated with a higher incidence of BMs, suggesting that these molecular subtypes may possess intrinsic neurotropic properties (Remon and Besse, 2018).

MiRNAs are short non-coding RNAs (∼22 nucleotides) that regulate gene expression post-transcriptionally, primarily by binding to complementary sequences within the 3′untranslated region of target mRNAs. In cancer, dysregulated miRNA expression contributes to tumor initiation, progression, and metastasis by modulating oncogenes, tumor suppressors, and key signaling pathways (Smolarz et al., 2022). Given that miRNAs regulate a wide range of genes involved in migration, invasion, adhesion, colonization, and epithelial–mesenchymal transition (EMT), they can be considered key molecular regulators within the metastatic cascade. Moreover, several studies have reported that miRNAs may con-tribute to the disruption of the blood–brain barrier and the establishment of pro-metastatic microenvironment (Souza et al., 2023; Solé and Lawrie, 2019; Kim et al., 2018). In LUAD-BM, limited therapeutic options, delayed diagnosis, and difficulties in accurate detection contribute to poor clinical outcomes (Souza et al., 2023; Kim et al., 2018; Tominaga et al., 2015).

Only a limited number of studies to date have identified dysregulated miRNAs in LUAD-BM (Remon et al., 2016; Koh et al., 2024; Zhang L. et al., 2022; Daugaard et al., 2017). Furthermore, it should be considered that expression patterns can vary across racial, ethnic, and geographic groups (Dluzen et al., 2016; Nassar et al., 2017; Rawlings-Goss et al., 2014). Identifying biomarkers that predict early metastatic dissemination and the establishment of a pro-metastatic microenvironment is therefore essential, as such molecules may offer novel therapeutic opportunities to prevent or limit the development of LUAD-BM.

In this study, we focused on characterizing the miRNA landscape in LUAD-BM using intraoperative tissue samples collected at the Department of Neurosurgery, Faculty of Medicine, University of Debrecen. Our objective was to identify specific miRNAs whose altered expression may contribute to metastatic progression and brain colonization, providing insights into the molecular mechanisms underlying LUAD-BM. Differentially expressed (DE) miRNAs between LUAD-BM and normal brain tissues were first identified through high-throughput next-generation sequencing (NGS) and subsequently validated in an extended cohort using real time quantitative polymerase chain reaction (RT-qPCR). To further investigate their functional relevance, pathway enrichment analyses were con-ducted to explore their roles in tumor progression and invasion. Integrating these findings with publicly available LUAD and normal lung miRNA-seq data allowed us to examine expression patterns across the normal lung–primary LUAD–LUAD-BM axis, high-lighting miRNAs potentially involved in metastatic spread and revealing novel candidates for diagnostic or therapeutic targeting.

## 2 Results

### 2.1 Clustering of RNA sequencing (RNA-seq) data and identification of DE miRNAs

**FIGURE 1:**
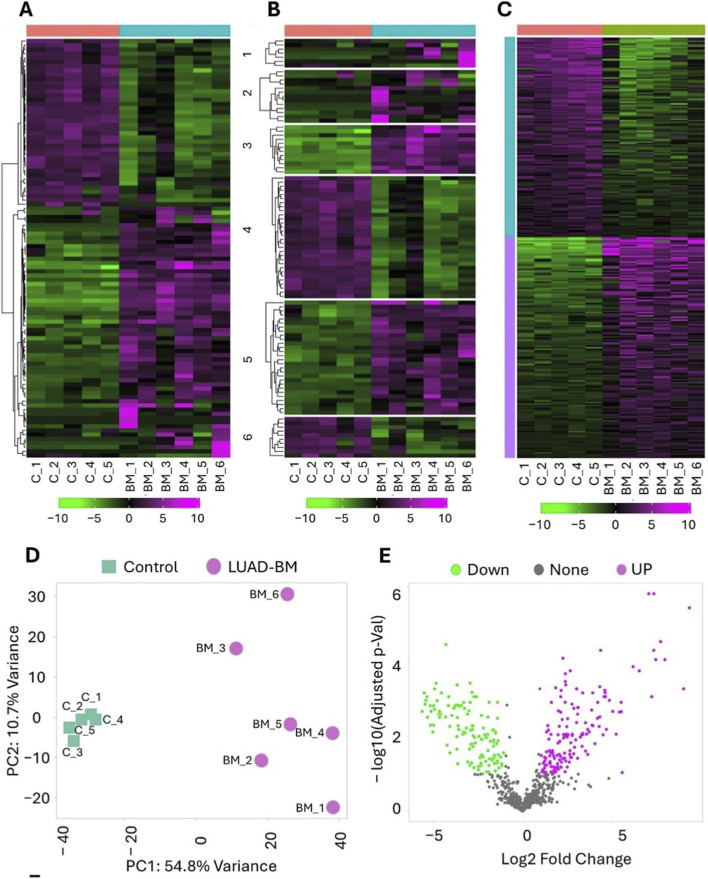
Hierarchical clustering, K-means clustering, principal component analysis (PCA), and differential expression analysis (DESeq2) of RNA-Seq data. **(A)** Heatmap of differentially expressed (DE) microRNAs (miRNAs) in LUAD brain metastasis (LUAD-BM) and control samples were created using iDEP 2.01. MiRNAs were ranked by their standard deviation across all samples, and hierarchical clustering was carried out for the top 100 miRNAs. Expression levels are represented by a color scale, with magenta indicating higher and green indicating lower relative expression. **(B)** K-means clustering was performed to identify different clusters in the dataset. The up- and downregulated miRNAs are labelled green and magenta, respectively. **(C)** The heatmap represents the upregulated (magenta) and downregulated (green) miRNAs. **(D)** The PCA plot illustrates the distribution of miRNA expression profiles in LUAD-BM samples (magenta) and control samples (green). **(E)** The volcano plot illustrates the differential expression of miRNAs in LUAD-BM compared to non-tumoros control samples, determined by DESeq2 analysis. MiRNAs exhibiting a log2Fold Change (FC) greater than 1 with a statistically significant p-value (<0.05) are highlighted in magenta, while those with a log2FC less than −1 and a p-value <0.05 are marked in green.

Brain tissue samples used for NGS were obtained from six LUAD-BM patients and from peritumoral, tumor-free regions of six lower-grade glioma patients, serving as control samples. We performed hierarchical clustering to compare the normalized RNA-seq datasets, revealing distinct differences in miRNA expression patterns between the LUAD-BM and control groups (Figure 1A). For the clustering analysis, miRNAs were ranked according to their standard deviation, and the 100 miRNAs with the largest expression changes were included in the analysis. With k-Means clustering, the top 100 miRNAs with the highest expression variability were explicitly divided into six groups based on their expression levels (Figure 1B).

Figure 1D presents the results of the principal component analysis as a scatter-plot. This technique enables dimensionality reduction of large datasets while preserving the overall variability of miRNA expression profiles. The plot clearly demonstrates a distinct separation between the metastatic and control groups along the PC1 axis, which ac-counts for 54.8% of the total variance. As expected, the tumor-free control samples form a tight cluster, whereas the metastatic samples—although they also cluster—exhibit significantly greater heterogeneity in expression. These findings, supported by both clustering and principal component analysis, confirm the distinctive miRNA expression profiles between the two groups, consistent with their underlying pathological characteristics.

Using the DESeq2 package within the iDEP 2.01 tool, a total of 118 significantly upregulated and 111 downregulated miRNAs were identified in LUAD-BM samples com-pared to controls (Supplementary Table S1). The analysis was performed using a threshold of false discovery rate (FDR) < 0.01 and fold change >2. The volcano plot (Figure 1E) and the heatmap (Figure 1C) show that the development of BM leads to a massive transcriptomic response.

### 2.2 Pathway enrichment analysis of DE miRNAs in LUAD-BM

**FIGURE 2:**
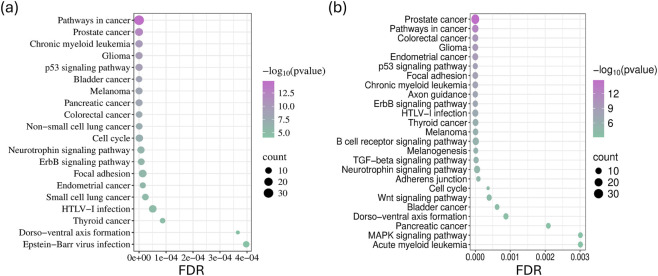
Kyoto Encyclopedia of Genes and Genomes (KEGG) pathway enrichment was analyzed for the 15 most upregulated **(a)** and downregulated **(b)** miRNAs, based on data from the miRNet tool. The dot sizes correspond to the number of genes associated with each KEGG pathway, and their significance is indicated by false discovery rate (FDR) values and the −log10 of the p-values.

We constructed miRNA–target gene interaction networks using the miRNet tool, followed by functional enrichment and pathway analysis using the Kyoto Encyclopedia of Genes and Genomes (KEGG) database. This approach enabled an independent evaluation of the enrichment results for miRNAs exhibiting the most significant expression changes in LUAD-BM samples. We considered the interaction networks of the 50 most upregulated and the 50 most downregulated miRNAs during the analysis. KEGG pathway analysis demonstrated that the targets of miRNAs overrepresented in LUAD-BM are involved not only in general tumor processes, the p53 signaling pathway, and cell cycle regulation, but also in pathways critical for migration, including focal adhesion, the ErbB signaling pathway, and the neurotrophin signaling pathway (Figure 2a). Furthermore, the upregulated miRNAs showed significant enrichment in the NSCLC pathway. Targets of downregulated miRNAs were also enriched in pathways such as the p53 signaling pathway, focal adhesion, adherens junction, neurotrophin signaling pathway, ErbB signaling pathway, cell cycle regulation, the transforming growth factor-beta signaling pathway, and Wnt signaling pathway (Figure 2b).

To minimize potential data noise, we analyzed the experimentally validated target genes of the five most upregulated and five most downregulated miRNAs using the miRTARGET web tool. Experimentally validated target genes were selected for each group based on statistical significance (p < 0.05). Approximately 38% of the targets of the top upregulated miRNAs were downregulated in LUAD, while about 30% showed increased expression. Conversely, among the targets of the most downregulated miRNAs, 25% exhibited reduced and 40% elevated expression in LUAD samples.

### 2.3 Validation of DE miRNAs by RT-qPCR

To validate the results obtained from NGS on a larger cohort, eight upregulated miRNAs–miR-200c-3p, miR-210-3p, miR-10a-5p, miR-130b-3p, miR-146b-5p, miR-503-5p, miR-196b-5p, and miR-3934-5p–and two downregulated miRNAs–miR-138-2-3p and miR-195-5p–were selected for RT-qPCR validation based on their log2 fold change (log2FC) and adjusted p-values (Table 1). RT-qPCR was performed to quantify the relative expression levels of the selected miRNAs, using miR-103a-3p as the reference miRNA. Validation was performed using total RNA isolated from 30 LUAD-BM and 30 control brain tissue samples.

**TABLE 1:** List of eight upregulated and two downregulated miRNAs selected for validation, including their corresponding log2Fold Change (log2FC) and adjusted P values.

Regulation	miRNA	LUAD-BM vs. Normal brain control (log2FC)	LUAD-BM vs. Normal brain Control (adj-pval)
Up	miR-200c-3p	9.24	<0.0001
Up	miR-210-3p	7.37	<0.0001
Up	miR-10a-5p	7.15	<0.001
Up	miR-130b-3p	4.57	<0.001
Up	miR-146b-5p	4.14	<0.01
Up	miR-503-5p	3.88	<0.001
Up	miR-196b-5p	3.54	<0.01
Up	miR-3934-5p	2.84	<0.01
Down	miR-138-2-3p	−2.1	<0.01
Down	miR-195-5p	−2.26	<0.05

**FIGURE 3:**
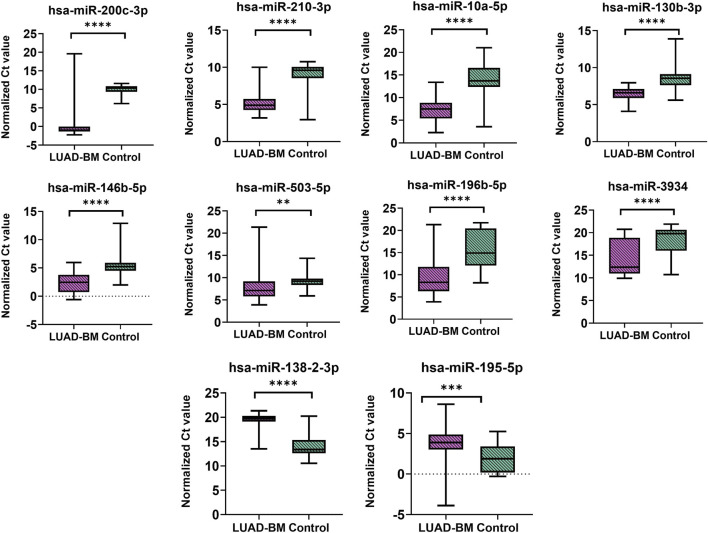
Statistical significance analysis of up- and downregulated miRNAs. Differences were analyzed using the Mann–Whitney U test. Color code: LUAD-BM, magenta; control, green. *p < 0.05; **p < 0.01; ***p < 0.001; ****p < 0.0001.

**FIGURE 4:**
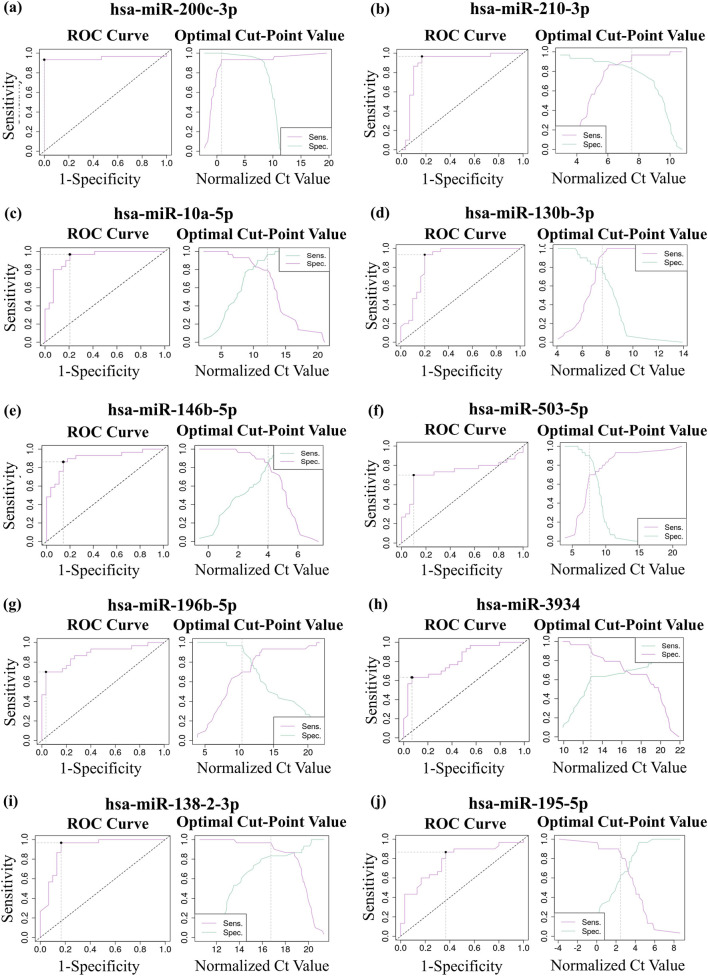
Receiver operating characteristic (ROC) analysis of up- and downregulated miRNAs. Analyses using the ROC curve analysis, distribution graph and sensitivity (Se) – specificity (Sp) curve of upregulated **(a)** miR-200c-3p (AUC: 0.95; optimal cut-point value: 0.82; Se: 0.93; Sp: 1); **(b)** miR-210-3p (AUC: 0.9; optimal cut-point value: 7.54; Se: 0.98. Sp: 0.83); **(c)** miR-10a-5p (AUC: 0.93; optimal cut-point value: 12.57; Se: 0.97; Sp:0.79); **(d)** miR-130b-3p (AUC: 0.87; optimal cut-point value: 7.58; Se: 0.93; Sp: 0.8); **(e)** miR-146b-5p (AUC: 0.89; optimal cut-point value: 4.08; Se: 0.86; Sp: 0.87); **(f)** miR-503-5p (AUC: 0.73; optimal cut-point value: 7.56; Se: 0.7; Sp: 0.9); **(g)** miR-196b-5p (AUC: 0.88; optimal cut-point value: 10.4; Se: 0.7; Sp: 0.97); **(h)** miR-3934-5p (AUC: 0.81; optimal cut-point value: 12.83; Se: 0.63; Sp: 0.93; **(i)** miR-138-2-3p (AUC: 0.91; Se: 0.97; Sp: 0.83); **(j)** miR-195-5p (AUC: 0.78; Se: 0.87; Sp: 0.63).

Based on the normalized Ct values obtained from RT-qPCR, statistical analysis using the Mann–Whitney U test confirmed that the expression levels of miR-200c-3p, miR-210-3p, miR-10a-5p, miR-130b-3p, miR-146b-5p, miR-503-5p, miR-196b-5p, and miR-3934-5p were significantly elevated in LUAD-BM samples compared to controls (Figure 3; Table 2). Conversely, the expression of miR-138-2-3p and miR-195-5p was found to be significantly reduced, further validating the NGS-based differential expression findings. To assess the diagnostic value of the selected miRNAs, receiver operating characteristic (ROC) curves and distribution graphs were generated, and the optimal threshold values, as well as sensitivity and specificity values, were determined (Figure 4; Table 2). The miR-200c-3p exhibited an area under the curve (AUC) of 0.95, accompanied by a sensitivity of 0.93 and specificity of 1 (Figure 4a). The miR-210-3p showed an AUC of 0.9, with sensitivity and specificity values of 0.98 and 0.83, respectively (Figure 4b). For miR-10a-5p, the AUC was 0.93, with sensitivity at 0.97 and specificity at 0.79 (Figure 4c). The miR-130b-3p had an AUC of 0.87, along with a sensitivity of 0.93 and a specificity of 0.8 (Figure 4d). Meanwhile, miR-146b-5p had both sensitivity and specificity at 0.86, with an AUC of 0.89 (Figure 4e). MiR-503-5p showed an AUC value of 0.73, with sensitivity and specificity measured at 0.7 and 0.9, respectively (Figure 4f). MiR-196b-5p had an AUC of 0.88, with a sensitivity of 0.7 and a specificity of 0.97, and for miR-3934-5p, the AUC was 0.81, with sensitivity at 0.63 and specificity at 0.93 (Figures 4g,h). For downregulated miR-138-2-3p and miR-195-5p, the AUC values were 0.91 and 0.78, the sensitivities were 0.97 and 0.87, and the specificity values were 0.83 and 0.63, respectively (Figures 4i,j). Based on available literature, we have summarized in Table 3 the diagnostic potential and prospective therapeutic applications of the miRNAs investigated in this study, highlighting their roles in lung cancer progression, drug resistance, and possible clinical relevance.

**TABLE 2:** Diagnostic performance of selected miRNAs based on ROC curve analysis. The table shows p values, area under the curve (AUC) with 95% confidence intervals (95% CI), optimal cut-off points, sensitivity, and specificity for each miRNA.

miRNA	p value	AUC	95% CI	Optimal cut-off point	Sensitivity	Specificity
miR-200c-3p	<0.00001	0.95	0.88–1.022	0.82	0.93	1
miR-210-3p	<0.00001	0.9	0.8–0.99	7.54	0.97	0.83
miR-10a-5p	<0.00001	0.93	0.86–0.99	12.57	0.97	0.79
miR-130b-3p	<0.00001	0.87	0.77–0.97	7.58	0.93	0.8
miR-146b-5p	<0.00001	0.89	0.82–0.98	4.08	0.86	0.86
miR-503-5p	<0.01	0.73	0.58–087	7.56	0.7	0.9
miR-196b-5p	<0.00001	0.88	0.74–0.95	10.4	0.7	0.97
miR-3934	<0.00001	0.81	0.71–0.92	12.83	0.63	0.93
miR-195-5p	<0.001	0.75	0.62–0.88	16.74	0.97	0.83
miR-138-2-3p	<0.00001	0.91	0.84–0.99	2.46	0.87	0.63

**TABLE 3:** Summary of the possible diagnostic and therapeutic applications of the miRNAs investigated in lung cancer based on published literature.

miRNA	Diagnostic potential	Therapeutic potential
miR-10a-5p	Elevated level is associated with aggressive disease and higher metastatic risk (Patnaik et al., 2011)	miR-10a inhibition may reverse cisplatin resistance in NSCLC (Sun et al., 2015) Targeting miR-10a could suppress tumor growth and invasion (via PTEN/AKT/ERK pathway) (Yu et al., 2015)
miR-146b-5p	May serve as a marker of recurrence and poor survival (Sang et al., 2025)	Inhibiting miR-146b-5p or PI3K/AKT signaling may reduce NSCLC growth and osimertinib resistance (Sang et al., 2025)
miR-200c-3p	Exhibits tumor-suppressive effects in NSCLC by inhibiting cell proliferation and migration (Lei et al., 2018)	Inhibits cell proliferation and migration in NSCLC by targeting LDHA (Lei et al., 2018) Inhibition of EMT via miR-200c-3p overexpression may overcome EGFR TKI resistance and promote NSCLC cell death (Wang et al., 2020)
miR-130b-3p	High expression correlates with poor overall survival and is associated with vascular and lymphatic invasion in NSCLC (Hirono et al., 2019)	Inhibition of APE1-mediated miR-33a/miR-130b regulation may restore DICER1 expression, reducing chemoresistance and invasiveness in lung cancer (Antoniali et al., 2022) Inhibition of DEPDC1 via exosomal miR-130b-3p may suppress NSCLC growth and migration, promote apoptosis, and block EMT (Lv et al., 2024) Inhibition of miR-130b or restoration of TIMP-2 may suppress NSCLC invasion by reducing MMP-2 activity (Hirono et al., 2019)
miR-503-5p	High miR-503-5p expression may serve as a biomarker for cisplatin resistance and angiogenesis in LUAD (Han and Wang, 2023)	Inhibition of miR-503-5p may overcome cisplatin resistance, suppress angiogenesis and EMT, and promote apoptosis in LUAD via upregulation of CTDSPL (Han and Wang, 2023) Inhibition of the JMJD2C/MALAT1/miR-503-5p/SEPT2 axis may suppress NSCLC progression by modulating histone methylation and downstream targets (Zhang et al., 2022b)
miR-210-3p	Upregulation of miR-210-3p in lung cancer tissues and cells may indicate poor prognosis in lung cancer (Chen et al., 2021)	Inhibition of PCGF3 via miR-210-3p antagomir may suppress lung cancer growth and metastasis by modulating Bax, Bcl-2, MMP-2, and MMP-9 expression Inhibition of miR-210-3p may suppress lung cancer progression and EMT while promoting apoptosis, partly via the USF1/PCGF3 pathway (Chen et al., 2021)
miR-3934-5p	TP53INP1 and miR-3934-5p levels may serve as biomarkers for lung cancer progression and cisplatin sensitivity (Ren et al., 2019)	Inhibition of miR-3934-5p may enhance cisplatin sensitivity, suppress proliferation, and promote apoptosis in NSCLC via upregulation of TP53INP1 (36)
miR-195-5p	Low miR-195 expression may indicate poor prognosis in lung cancer (Liu et al., 2015)	Inhibition of CHEK1 via miR-195 overexpression may suppress NSCLC growth, migration, and invasion, improving prognosis (Liu et al., 2015) Inhibition of FGF2 via miR-195-5p overexpression may enhance cisplatin sensitivity and suppress migration, invasion, and EMT in NSCLC (Wang et al., 2021)

### 2.4 Comparative analysis of miRNA profiles in LUAD-BM and LUAD tissues

To investigate whether the experimentally validated differentially expressed (DE) miRNAs identified in LUAD brain metastases are also altered in primary lung tumors, differential expression analysis was performed using LUAD-BM samples from our cohort (n = 6). For comparison, miRNA expression profiles of primary LUAD (n = 20) and normal lung control samples (n = 15) were obtained from The Cancer Genome Atlas (TCGA) database and used to assess expression patterns along the normal lung–primary LUAD–LUAD-BM axis. The analysis indicated significant downregulation of miR-200c-3p (log2FC = −1.30, *p* < 0.05), while significant upregulation of miR-146b-5p (log2FC = 4.34, *p* < 0.0001) and miR-3934-5p (log2FC = 2.60, *p* < 0.0001) in LUAD-BM in comparison to primary LUAD samples. However, these miRNAs did not show differential expression in primary LUAD compared to normal lung controls. The analysis further demonstrated that miR-10a-5p (log2FC = −1.19, *p* < 0.01) was significantly downregulated in primary LUAD samples relative to normal lung controls, with an additional de-crease in expression observed in LUAD-BM compared to primary LUAD (log2FC = −2.17, *p* < 0.0001). Conversely, miR-210-3p (log2FC = 4.97, *p* < 0.0001) and miR-130b-3p (log2FC = 1.11, *p* < 0.01) were significantly upregulated in primary LUAD samples relative to normal lung controls, and their expression levels increased further in LU-AD-BM compared to primary LUAD (miR-210-3p: log2FC = 1.93, *p* < 0.0001; miR-130b-3p: log2FC = 3.24, *p* < 0.0001). No significant differences in expression were observed for miR-503-5p and miR-195-5p in the LUAD-BM versus LUAD comparison. However, miR-503-5p (log2FC = 2.25, *p* < 0.0001) was significantly upregulated, while miR-195-5p (log2FC = −3.32, *p* < 0.0001) was significantly downregulated in primary LUAD samples compared to normal lung controls. Moreover, miR-138-2-3p is not ex-pressed in lung tissue, which explains why no differences were observed along the nor-mal lung–LUAD–LUAD-BM axis. However, its expression was found to be downregulated in LUAD-BM compared to normal brain tissue. These results indicate that among the ten validated miRNAs that were significantly dysregulated in LUAD-BM compared with control brain tissue samples, miR-200c-3p, miR-146b-5p, and miR-3934-5p showed significant expression differences between primary LUAD and LUAD-BM, while miR-10a-5p, miR-210-3p, and miR-130b-3p exhibited stepwise dysregulation from normal lung to primary LUAD and further to LUAD-BM (Table 4). These results may reflect their role in metastatic progression.

**TABLE 4:** Comparison of the expression levels of 10 DE miRNAs, including their respective log2FC and adj-p-values. The analysis was performed by first comparing LUAD samples with normal lung samples, followed by a comparison of LUAD-BM samples with LUAD controls from the TCGA database.

miRNA	LUAD vs. Normal lung control (log2FC)	LUAD vs. Normal lung control (adj-pval)	LUAD-BM vs. LUAD (log2FC)	LUAD-BM vs. LUAD (adj-pval)
miR-196b-5p	3.53	<0.0001	−3.14	<0.0001
miR-10a-5p	−1.19	<0.01	−2.17	<0.0001
miR-200c-3p	0.36	ns	−1.30	<0.05
miR-503-5p	2.45	<0.0001	0.48	ns
miR-195-5p	−3.32	<0.0001	0.91	ns
miR-210-3p	4.97	<0.0001	1.93	<0.0001
miR-3934-5p	−0.89	<0.05	2.60	<0.0001
miR-130b-3p	1.11	<0.01	3.24	<0.0001
miR-146b-5p	−0.85	<0.05	4.34	<0.0001
miR-138-2-3p	0	ns	0	ns

## 3 Discussion

Lung cancer represents the most prevalent cause for cancer-related mortality world-wide; within this, approximately 85% of all cases are diagnosed with NSCLC (Zhou et al., 2021). A considerable proportion of patients with NSCLC develop BM that significantly worsen prognosis and pose unique therapeutic challenges, especially among patients with LUAD. Response rates to monotherapy with PD-1 or PD-L1 inhibitors among those with BMs are generally low (Kim et al., 2020). It can be explained by the unique immune microenvironment of the central nervous system, restricted drug penetrance across the blood–brain barrier, and the clonal selection and evolution of those groups of tumor cells that tend to migrate to the brain (Tsakonas et al., 2022). Recently, miRNAs have emerged as critical regulators of gene expression and key players in cancer biology. These small, non-coding RNA molecules can influence multiple oncogenic processes, including proliferation, invasion, metastasis, and therapy resistance (Hudson et al., 2024). In this study, we aimed to identify miRNAs that are DE in LUAD-BM tissue samples compared to control brain and primary LUAD samples, with the goal of uncovering candidates potentially involved in the development of BM originating from LUAD. Further-more, we explored their potential utility as biomarkers for predicting the risk of BM in LUAD patients.

KEGG pathway analysis was performed using the 50 most upregulated and 50 most downregulated miRNAs identified by miRNA NGS in the LUAD-BM versus control brain tissue comparison. The predicted target genes of these dysregulated miRNAs showed a strong association with cancer-related pathways, highlighting their potential involvement in tumor progression and metastasis. For example, aberrant expression of focal adhesion molecules that act as mechanosensors mediating bidirectional communication between the cell and its microenvironment could lead to enhanced invasion and migration capacity of the tumor cells (Yayan et al., 2024). It has been shown that the application of integrin inhibitors and E-cadherin up-regulators during therapy leads to a 70% reduction of invasion (Riley et al., 2019). Furthermore, neurotrophin signaling pathways play a significant role in tumor development. Specifically, nerve growth factor and brain-derived neurotrophic factor were reported to stimulate tumor cell proliferation, survival, migration, and/or invasion and favor tumor angiogenesis (Chopin et al., 2016).

**FIGURE 5:**
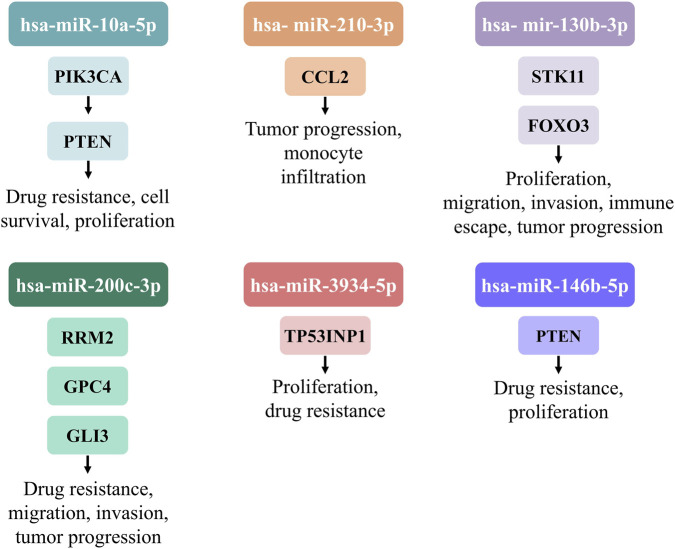
Schematic representation of experimentally validated target genes of miR-10a-5p, miR-210-3p, miR-130b-3p, miR-3934-5p, miR-200c-3p and miR-146-5p, illustrating their functional roles and biological implications in LUAD/NSCLC, based on literature data.

Our RT-qPCR validation confirmed that the expression levels of miR-200c-3p, miR-210-3p, miR-10a-5p, miR-130b-3p, miR-146b-5p, miR-503-5p, miR-196b-5p, and miR-3934-5p were significantly upregulated, while miR-138-2-3p and miR-195-5p were significantly downregulated in LUAD-BM tissue samples compared to control brain tis-sue. In addition to identifying dysregulated miRNAs in LUAD-BM tissue samples compared to brain tissue controls, we performed differential expression analysis on 15 normal lung tissue samples, 20 primary LUAD samples–downloaded from the TCGA database and selected from European populations–and our six LUAD-BM samples (Table 4). Furthermore, based on literature data, we analyzed the functions of the target genes of the selected miRNAs, focusing on those previously shown to be associated with migratory processes in LUAD/NSCLC (Table 5; Figure 5).

**TABLE 5:** Experimentally validated target genes of miR-10a-5p, miR-210-3p, miR-130b-3p, miR-3934-5p, miR-200c-3p and miR-146-5p together with their associated biological functions and functional effects in LUAD/NSCLC, based on literature data.

miRNA	Target	Biological function	Functional effect in LUAD/NCSLC	Reference
miR-10a-5p	PIK3CA	Regulates AKT/mTOR signaling, cell growth, survival	Suppresses PI3K/AKT pathway; modulates cisplatin resistance	Huang et al. (2020)
miR-10a-5p	PTEN	Negative regulator of PI3K/AKT signaling, cell proliferation, migration	Downregulation promotes proliferation, migration, invasion via AKT/ERK activation	Yu et al. (2015)
miR-210-3p	CCL2	Regulates macrophage recruitment and polarization under hypoxia	High levels reduce monocyte infiltration, and promote tumor progression	Arora et al. (2024)
mir-130b-3p	STK11	Regulates cell proliferation, migration, invasion, and immune escape	Upregulation promotes the proliferation, migration, invasion, and immune escape	Chen et al. (2023)
mir-130b-3p	FOXO3	Regulation of Keap1/NFE2L2/TXNRD1 signaling and tumor progression	Upregulation promotes lung cancer progression	Guo et al. (2021)
miR-200c-3p	RRM2	Regulates DNA synthesis and cell proliferation	Downregulation promotes DDP resistance and tumor progression	Liu et al. (2022)
miR-200c-3p	GPC4	Regulates HS3ST1-mediated glycolysis, tumor growth and metastasis	Downregulation enhances glycolysis, cell proliferation, migration, invasion and tumor growth	Ji et al. (2024)
miR-200c-3p	GLI3	Regulate cell cycle and proliferation	Downregulation promotes proliferation, invasion, and inhibits apoptosis	Yi et al. (2024)
miR-3934-5p	TP53INP1	Regulates cell proliferation, and apoptosis	Upregulation leads to increased proliferation, decreased apoptosis, and enhanced DDP resistance	Ren et al. (2019)
miR-146b-5p	PTEN	Regulates PI3K/AKT signaling pathway, proliferation	Upregulation promotes NSCLC proliferation and osimertinib resistance	Sang et al. (2025)

Based on differential expression analysis conducted on the above-mentioned tissue samples miR-200c-3p expression was significantly decreased, whereas miR-146b-5p and miR-3934-5p expression was significantly increased in LUAD-BM samples compared to primary LUAD samples, while no detectable differences were observed between primary tumors and normal lung tissue. These findings suggest that the expression changes of these miRNAs are specifically associated with the development of secondary brain tumors. The upregulation of miR-146b-5p and miR-3934-5p observed in the LUAD-BM versus primary LUAD comparison is consistent with our findings in the LUAD-BM versus normal brain comparison, where we also detected upregulation.

According to several studies, miR-200c may exert contrasting regulatory effects depending on the cancer type, tumor microenvironment, and even the specific genetic background of the tumor cells (Guo et al., 2025; Klicka et al., 2022). However, in LUAD tissue samples compared to normal lung tissue, miR-200c has generally been identified as a tumor suppressor (Liu et al., 2017). It has been reported that all members of the miR-200 family are highly expressed in lung tissue but are barely detectable in the brain (Teplyuk et al., 2012; Liang et al., 2007). These data are consistent with our results, as our sequencing data indicated that normal brain expresses miR-200c-3p at significantly lower levels than normal lung. This provides an explanation for the observed relative increase of miR-200c-3p in LUAD-BM samples compared to normal brain, despite the downregulation detected in primary LUAD. MiR-200c proved to be involved in EMT in LUAD, which is a hallmark of metastasis that facilitates detachment from the primary tumor, enhanced motility, and invasion. (Gregory et al., 2008). Lei et al. demonstrated that miR-200c exhibits tumor-suppressive effects in NSCLC by inhibiting cell proliferation and migration through targeting lactate dehydrogenase A, representing a potential therapeutic target (Lei et al., 2018). Moreover, miR-200c-3p contributes to EGFR TKI sensitivity by regulating the EMT process (Wang et al., 2020).

Interestingly, both tumor-suppressive and oncogenic roles of miR-146b-5p have been reported in NSCLC, highlighting its context-dependent regulatory function. For example, Sang et al. demonstrated its significantly elevated level in NSCLC tissue samples coupled with worse prognosis. Furthermore, they proved that methyltransferase 16 mediated miR-146b-5p m6A modification led to the induction of cell proliferation and osimertinib resistance via activation of the phosphatidylinositol 3-kinase/AKT signaling pathway, and modification of miR-146b-5p may promote osimertinib resistance (Sang et al., 2025). These data, consistent with our results, support the notion that inhibition of miR-146b-5p overexpression could serve as a potential therapeutic strategy against LUAD-BM and the suppression of chemotherapy resistance.

MiR-3934-5p remains poorly characterized, as it has been rarely investigated in previous studies. Ren et al. highlighted the possible therapeutic potential of miR-3934-5p in A549 cell line, showing that inhibiting miR-3934-5p can restore tumor protein P53 inducible nuclear protein 1 expression and make LUAD cells more responsive to chemotherapeutic agents such as cisplatin. MiR-3934-5p upregulation enhances proliferation, inhibits apoptosis, and confers DDP resistance in lung cancer (Ren et al., 2019). We propose that inhibition of miR-3934-5p could possess possible therapeutic potential in overcoming therapy resistance.

The analysis revealed a significant downregulation of miR-10a-5p in primary LUAD samples compared to normal lung controls, with further decreased expression observed in LUAD-BM relative to primary LUAD. In contrast, miR-210-3p and miR-130b-3p were significantly upregulated in primary LUAD compared to normal lung, and their expression levels increased further when LUAD-BM was compared to primary LUAD. These results suggest that the expression changes of these miRNAs may be closely associated with the progression of metastasis.

The dual role of miR-10a-5p as both a tumor suppressor and an oncogene has been reported in several cancer types, including breast, bladder, ovarian, and gastric cancers (Hu et al., 2019; Nakayama et al., 2013; Gao et al., 2024; Zaravinos et al., 2012; Xiao et al., 2014; Ma et al., 2007; Moriarty et al., 2010; Wang et al., 2015; Singh et al., 2025). Additionally, previous studies have reported that miR-10a-5p acts as a tumor suppressor in gliomas by inhibiting glioma cell proliferation and migration via downregulation of tumor suppressor candidate 7 (Wang et al., 2023). However, only a few studies have investigated the role of miR-10a-5p in NSCLC. Yu et al. proved that upregulation of miR-10a tends to induce metastasis formation of lung cancer through the regulation of the phosphatase and tensin homolog/AKT/mitogen-activated protein kinase signaling pathway (Yan et al., 2015). Furthermore, silencing miR-10a reverses cisplatin resistance in human lung cancer cell lines through the transforming growth factor-beta/Smad2/signal transducer and activator of transcription 3/signal transducer and activator of transcription 5 path-way, thereby indicating a possible therapeutic relevance (Sun et al., 2015). Contradictory results have been reported regarding miR-10a-5p in studies of LUAD. These discrepancies may be attributed to differences in expression across racial, ethnic, and geographic populations. Contradictory results have been reported regarding miR-10a-5p in studies of LUAD. These discrepancies may be attributed to differences in expression across racial, ethnic, and geographic populations.

In the development and growth of metastatic brain tumors, the brain microenvironment, composed mainly of the astrocytes, plays an important role (Alsidawi et al., 2014). According to the results of several studies, miR-210 directly induces a change in the brain microenvironment. For example, according to the analysis of Camacho et al., miR-210 isolated from exosomes of metastatic brain cells with breast cancer origin was the only significantly over-expressed miRNA when compared to parental breast cancer cells (Camacho et al., 2013). In LUAD, elevated miR-210 supports survival in the brain niche by modulating mitochondrial metabolism and reducing oxidative stress (Puisségur et al., 2011). In the context of lung cancer, inhibition of miR-210-3p in A549 cells resulted in reduced migration, invasion capacity, and cell viability, while significantly increasing the rate of apoptosis (Chen et al., 2021). According to another study, a three-miRNA (miR-210, miR-214, and miR-15a) based signature was able to predict BM in LUAD patients with 91.4% accuracy (Zhao et al., 2018). It has also been reported that high levels of miR-210-3p promote tumor progression by modulating C-C motif chemokine ligand 2 mediated macrophage recruitment and polarization under hypoxic conditions (Arora et al., 2024). Furthermore, Chen et al. demonstrated that silencing miR-210-3p may represent a promising approach for lung cancer treatment, as it can inhibit tumor growth, invasion, and metastasis by modulating the polycomb group ping finger 3/upstream transcription factor 1 axis, while also affecting apoptosis, angiogenesis, and EMT (Chen et al., 2021). Daugaard et al. also reported the upregulation of miR-210-3p and found that it was significantly associated with the development of distant metastases (Daugaard et al., 2017). These results, consistent with our findings, support the notion that miR-210-3p may serve as a potential therapeutic target to prevent the development of LUAD-BM.

Previous clinical studies have demonstrated that overexpression of miR-130b is associated with aggressive tumor phenotypes and poor prognosis across several cancer types, including glioma (Alsidawi et al., 2014). Moreover, elevated miR-130b expression has been significantly correlated with the formation of distant metastases in both colon and lung cancers (Alsidawi et al., 2014). In LUAD, Kim et al. reported that miR-130b overexpression was linked to higher histological grade, lymph node metastasis, and lymphovascular invasion, further supporting its role in tumor progression and metastatic potential (Kim et al., 2021). It has also been reported that upregulation of miR-130b-3p promotes lung cancer progression by regulating serine/threonine kinase 11 mediated proliferation, migration, invasion, and immune escape, as well as FOXO3-dependent kelch like ECH associated protein 1/NFE2 like BZIP transcription factor 2/thioredoxin reductase 1 signaling (Chen et al., 2023; Guo et al., 2021). Moreover, miR-130b-3p is considered a possible therapeutic target in NSCLC, as exosomal miR-130b-3p has been shown to serve as a potential predictive marker and therapeutic target by suppressing NSCLC growth, migration, and EMT, and promoting apoptosis through inhibition of DEP Domain Containing 1 (Lv et al., 2024). Furthermore, inhibition of miR-130b or restoration of TIMP metallopeptidase inhibitor 2 may reduce NSCLC invasion by decreasing Matrix Metallopeptidase 2 activity (Hirono et al., 2019; Frydrychowicz et al., 2023). These data, consistent with our findings–showing that changes in miR-130b-3p expression can be detected even in primary tumor–suggest that targeting this miRNA may play a significant role in inhibiting the metastatic process from its initial phases.

Furthermore, no significant differences in expression of miR-503-5p and miR-195-5p were observed between LUAD-BM and primary LUAD. However, miR-503-5p was significantly upregulated, whereas miR-195-5p was significantly downregulated in primary LUAD compared to normal lung controls.

MiR-503-5p shows association with regulation of the development of different cancer types like LUAD and additionally, it accelerates metastasis and angiogenesis formation in tumors, too (Fei et al., 2020). Recent studies have reported that miR-503-5p plays a role in mediating chemotherapy resistance in various tumor types (Park and Kim, 2019). Han et al. demonstrated that elevated levels of it were associated with cisplatin resistance and angiogenesis of LUAD cells via regulation of CTD small phosphatase like gene expression, suggesting that miR-503-5p may serve as a potential therapeutic target (Han and Wang, 2023). Moreover, Zhang et al. demonstrated that inhibition of the JMJD2C/metastasis associated lung adenocarcinoma transcript 1/miR-503-5p/Septin 2 axis may suppress NSCLC progression by modulating histone methylation (Zhang J. et al., 2022).

Deregulation of miR-195-5p has been reported in multiple cancers. Depending on the specific cancer type, it can play an oncogenic or tumor-suppressive role as a posttranscriptional regulator (Mohan et al., 2024). Long et al. detected the significant downregulation of miR-195-5p both in LUAD tissue samples and in lung cancer cell lines (H1299, A549) in comparison with the control samples. Additionally, they demonstrated that elevated expression of miR-195-5p can induce inhibition of lung cancer cell proliferation, migration, and invasion, leading to reduced tumor growth and metastasis formation of lung cancer via targeting forkhead box K1 (Long and Wang, 2020). Other studies proved the tumor suppressor activity of miR-195-5p in NSCLC through the direct regulation of MYB, insulin-like growth factor 1 receptor, hepatoma-derived growth factor or checkpoint kinase 1 (Liu et al., 2015; Guo et al., 2014; Wang et al., 2014; Yongchun et al., 2014). It was further demonstrated that miR-195 is associated with a better prognosis in lung cancer, while targeting Fibroblast Growth Factor 2 can enhance chemosensitivity in A549/DDP cells (Liu et al., 2015; Wang et al., 2021).

A limited number of studies have focused on the miRNA expression differences in LU-AD-BM compared to primary LUAD. Zhang et al. compared the miRNA expression pro-files of LUAD samples without BM to those with BM to identify potential biomarkers and mechanisms involved in metastasis. They identified 20 dysregulated miRNAs using next-generation RNA sequencing in an Asian population (Zhang L. et al., 2022). In another study, the authors identified a 25-miRNA signature based on the mRNA profiles of LUAD patients with and without BMs, based on their tissue mRNA profiles using machine learning–based prediction (Koh et al., 2024). Remon et al. compared expression patterns between EGFR-mutant NSCLC samples without BMs and those with confirmed BMs (Remon et al., 2016).

Tsakonas et al. performed matched analyses to reveal a unique miRNA signature by comparing primary NSCLC samples to brain metastatic samples. The study reported downregulation of miR-129-2-3p, miR-124-3p, miR-219a-2-3p, miR-219a-5p, and miR-9-5p, and upregulation of miR-142-3p, miR-150-5p, miR-199b-5p, miR-199a-3p, and miR-199a-5p (Tsakonas et al., 2022). The heterogeneity observed in miRNA expression patterns is likely influenced by population-specific genetic factors that affect baseline miRNA levels. Consequently, expression patterns may vary across racial, ethnic, and geographic groups (Dluzen et al., 2016; Nassar et al., 2017; Rawlings-Goss et al., 2014). Therefore, geographic and ethnic context should be considered a key factor when assessing the clinical utility of miRNAs.

To the best of our knowledge, this is the first study to simultaneously compare the miRNA expression profiles of LUAD-BM tissues with normal brain tissue, primary LU-AD, and normal lung. This approach allows mapping of expression changes along the transition from normal lung to primary LUAD and LUAD-BM. Additionally, this is the first study specifically focusing on LUAD that utilized brain tissue samples to investigate the expression profiles of LUAD-BM. Furthermore, we identified for the first time the dysregulation of miR-10a-5p, miR-200c-3p, miR-3934-5p, miR-130b-3p, miR-146b-5p, miR-503-5p, and miR-195-5p in LUAD-BM human samples that could play an important role in the development of BM with LUAD origin. Our findings provide initial insights in-to the role of these miRNAs; however, they should be regarded as preliminary, and additional research is required to determine their diagnostic and therapeutic implications.

## 4 Materials and methods

### 4.1 Clinical sample collection and patient inclusion/exclusion criteria

For NGS analysis, six LUAD-BM and six control brain tissue samples were included. The miRNA expression profiles were previously published and retrieved from the Gene Expression Omnibus database (accession numbers GSE284777 and GSE244332) (Torner et al., 2025; Géczi et al., 2025). All samples were obtained from surgical resections. In addition, 30 patients from the same cohort in each group were included for validation. LUAD-BM brain tissue samples and peritumoral brain tissue samples from patients with low-grade glioma (WHO grade I–II), used as controls, were collected during surgery. Patients were identified between 2010 and 2024 at the Department of Neurosurgery, University of Debrecen, Faculty of Medicine. The samples were flash frozen immediately after re-section and stored at −80 °C until analysis. The samples were obtained from the right frontal or temporal lobes, and the diagnoses were confirmed by histopathological examination. None of the patients received chemotherapy or radiotherapy prior to surgery. LUAD-BM and control groups were age-matched, with an average age of 61.46 years in the LUAD-BM cohort, and the gender distribution was balanced across both groups (Table 6). The study was approved by the Scientific and Research Ethics Committee of the Medical Research Council of the Ministry of Health, Budapest, Hungary (ETT TUKEB; project identification code: IV/1753-/2021/EKU) and was conducted in accordance with the Declaration of Helsinki, and all participants signed a consent form.

**TABLE 6:** Summary of the characteristics of the 6 LUAD-BM and 6 normal brain control patients selected for NGS analysis.

Characteristic	Gender	Age	Immunohistochemical characteristics
LUAD-BM_1	M	67	CK7 +, TTF-1+, CD × 2 −
LUAD-BM_2	M	71	CK7 +, TTF-1+, CD × 2 −
LUAD-BM_3	M	73	CK7 +, TTF-1+, CD × 2 −
LUAD-BM_4	F	66	CK7 +, TTF-1+, CD × 2 −
LUAD-BM_5	F	71	CK7 +, TTF-1+, CD × 2 −
LUAD-BM_6	F	59	CK7 +, TTF-1+, CD × 2 −
Controll_1	M	70	-
Controll_2	M	52	-
Controll_3	M	52	-
Controll_4	F	71	-
Controll_5	F	80	-
Controll_6	F	61	-

### 4.2 Small RNA library preparation and NGS

To investigate the miRNA expression profiles of LUAD-BM and control brain tissue samples, small-RNA-seq analysis was conducted on 12 selected brain specimens in collaboration with the Genomic Medicine and Bioinformatics Core Facility (Department of Biochemistry and Molecular Biology, Faculty of Medicine, University of Debrecen). Library preparation was performed using the NEBNext Multiplex Small RNA Prep Set for Illumina (Molina et al., 2008; Herbst et al., 2018; Lukas et al., 2014; Sperduto et al., 2020; Le Rhun et al., 2021; Najjary et al., 2023; Remon and Besse, 2018; Smolarz et al., 2022; Souza et al., 2023; Solé and Lawrie, 2019; Kim et al., 2018; Tominaga et al., 2015; Remon et al., 2016; Koh et al., 2024; Zhang L. et al., 2022; Daugaard et al., 2017; Dluzen et al., 2016; Nassar et al., 2017; Rawlings-Goss et al., 2014; Zhou et al., 2021; Kim et al., 2020; Tsakonas et al., 2022; Hudson et al., 2024; Yayan et al., 2024; Riley et al., 2019; Chopin et al., 2016; Guo et al., 2025; Klicka et al., 2022; Liu et al., 2017; Teplyuk et al., 2012; Liang et al., 2007; Gregory et al., 2008; Lei et al., 2018; Wang et al., 2020; Sang et al., 2025; Ren et al., 2019; Hu et al., 2019; Nakayama et al., 2013; Gao et al., 2024; Zaravinos et al., 2012; Xiao et al., 2014; Ma et al., 2007; Moriarty et al., 2010; Wang et al., 2015; Singh et al., 2025) kit (New England BioLabs, Ipswich, MA, United States). The integrity and quality of total RNA were assessed with the Eukaryotic Total RNA Nano Assay on an Agilent BioAnalyzer (Agilent Technologies, Santa Clara, CA, United States). For library construction, 1 µg of total RNA with a RIN value above 7 was used. Fragment size distribution and library molarity were verified on the Agilent BioAnalyzer DNA1000 chip (Agilent Technologies, Santa Clara, CA, United States). Sequencing was carried out on the Illumina NextSeq 500 platform (Illumina, San Diego, CA, United States) generating 50 bp single-end reads. Raw sequence data were aligned to the human reference genome (GRCh38) using the Novoalign algorithm, optimized for short reads and miRNA-seq data. Prior to alignment, 3′ adapter sequences were removed using Novoalign’s built-in adapter-trimming function. Both default Illumina adapter sequences and custom adapter sequences were specified to ensure accurate trimming. Reads shorter than 17 nucleotides after adapter removal were filtered out as low-quality sequences. A homopolymer filter was applied to further remove low-quality reads. During mapping to the human reference genome, only one mismatch per read was allowed, and in the case of multiple mapping locations, the alignment with the best score was retained while other potential alignments were discarded. Subsequent data processing was performed with StrandNGS v4.0 software (www.strand-ngs.com, accessed on 3 March 2021). Normalization of expression data was conducted using the DESeq algorithm, and DE miRNAs were identified by applying a moderated t-test.

### 4.3 Differential expression analysis

MiRNA expression data were analyzed using the iDEP 2.01 web-based platform (https://bioinformatics.sdstate.edu/idep/(accessed on 19 April 2024)). Hierarchical clustering was conducted by applying a Z-score cutoff of 3 to filter the data, and K-Means clustering was subsequently performed on the 100 most variable miRNAs to explore expression patterns. Principal component analysis was also utilized to visualize sample distribution based on expression profiles. Differential gene expression analysis was carried out using the DESeq2 software package, integrated within the iDEP 2.01 pipeline. The differential gene expression analysis employed an FDR threshold of 0.01 and a minimum FC of 2 to identify DE miRNAs. MiRNAs were considered significantly upregulated if their FC was ≥2 with an FDR ≤0.05, while miRNAs with an FC ≤ −2 and an FDR ≤0.05 were classified as significantly downregulated (Ge et al., 2018).

### 4.4 Bioinformatical analysis

To identify experimentally validated target genes of the DE miRNAs, the miRNet platform (https://www.mirnet.ca (accessed on 27 June 2024)) was utilized in combination with the miRTarBase v9.0 database (https://awi.cuhk.edu.cn/∼miRTarBase/miRTarBase_2025/php/index.php (accessed on 15 September 2021)) for interaction network construction.

KEGG pathway enrichment analysis was performed using the integrated KEGG resources available within the miRNet environment, and pathways with a p-value below 0.05 were considered statistically significant. Shared target genes for the validated miRNAs were also identified based on the miRTarBase v9.0 dataset.

### 4.5 Tissue disruption, RNA extraction, and RT-qPCR-based validation of DE miRNAs

For total RNA isolation, 30 mg of flash-frozen tissue per sample was dissected on ice. Tissue disruption and homogenization were carried out using a MagNa Lyser instrument (Roche Ltd., Basel, Switzerland) using Qiazol lysis reagent and stainless-steel beads. MiRNA-enriched total RNA was extracted using the miRNeasy Mini Kit (Qiagen, Hilden, Germany), following the manufacturer’s protocol. The concentration and purity of the extracted RNA were assessed with a Nanodrop spectrophotometer (Thermo Scientific, Waltham, MA, United States). For the validation phase, total RNA isolated from 30 control and 30 LUAD-BM patient samples was reverse-transcribed into cDNA using the miRCURY LNA RT Kit (Qiagen, Hilden, Germany) following the manufacturer’s protocol, with incubation at 42 °C for 60 min and a final step at 95 °C for 5 min to terminate the reaction. The expression levels of miR-196b-5p, miR-130b-3p, miR-200c-3p, miR-210-3p, miR-503-5p, miR-195-5p, and miR-138-2-3p were quantified by real-time PCR using the LightCycler® 96 System (Roche Ltd., Pleasanton, CA, United States) and the miRCURY LNA SYBR Green PCR Kit (Qiagen, Hilden, Germany), according to the manufacturer’s instructions.

PCR conditions were set as follows: initial denaturation at 95 °C for 2 min, followed by 45 cycles of denaturation at 95 °C for 10 s, and annealing/extension in combination at 56 °C for 60 s. Melting curves were created by fluorescent measurements in three steps (95 °C for 20 s, 40 °C for 20 s, and 85 °C for 1 s), followed by a final cooling step at 37 °C for 30 s. Relative miRNA expression levels were calculated using the comparative cycle threshold (ΔΔCt) method, with miR-103a-3p serving as the internal reference (ΔCt = Ct_target miRNA_ − Ct_miR-103a-3p_) (Veryaskina et al., 2022). All reactions were performed in triplicate to ensure reproducibility.

### 4.6 Statistical analysis

Data normality was assessed using the Shapiro–Wilk test. Statistical analyses were performed using the non-parametric Mann–Whitney U test in GraphPad Prism 7, and differences in miRNA expression level were considered statistically significant at *p* < 0.05. ROC curve analysis was carried out using the easyROC 1.3.1 web-based tool [http://biosoft.erciyes.edu.tr/app/easyROC/(accessed on 25 July 2016)] and 95% confidence intervals were calculated using the DeLong method. The AUC was calculated to evaluate diagnostic performance, and the optimal cut-off value was selected based on the balance between sensitivity and specificity. Optimal cut-off was determined using Youden’s index.

### 4.7 TCGA-based comparative analysis

In addition, miRNA expression and corresponding clinical data from 20 LUAD and 15 lung control samples were obtained from TCGA database [http://cancergenome.nih.gov/(accessed on 7 May 2025)]. Only cases meeting the following criteria were included in the analysis: i) histopathologically confirmed LUAD; ii) absence of any other malignancy; iii) age between 50–75 years; iv) classification as White as defined by the TCGA race variable; v) tumor grade II–IV. The miRNA expression profiles of normal lung tissue samples were obtained from adjacent, non-tumorous lung tissues. Both miRNA expression data and clinical data are publicly available and open access (Table 7).

**TABLE 7:** Summary of the characteristics of the 20 LUAD and 15 normal lung control patients selected from TCGA database.

Characteristic	Gender	Age	TNM staging
LUAD1	F	59	N2
LUAD2	F	61	T2, N0
LUAD3	F	58	N0
LUAD4	F	54	N0
LUAD5	M	59	T2, N0
LUAD6	M	59	T2, N0
LUAD7	M	56	T3, N0
LUAD8	M	70	T3, N0
LUAD9	M	73	T3, N0
LUAD10	M	69	T2b, N0
LUAD11	F	70	T2b, N2
LUAD12	F	56	T1, N2
LUAD13	M	59	T2, N2
LUAD14	F	53	T1, N2
LUAD15	F	74	T1b, N2
LUAD16	F	58	T1, N2
LUAD17	M	72	T3, N2
LUAD18	M	70	T1, N2
LUAD19	M	69	T2, N2
LUAD20	M	61	T2, N2
Normal lung1	F	68	-
Normal lung2	F	52	-
Normal lung3	M	58	-
Normal lung4	F	52	-
Normal lung5	M	60	-
Normal lung6	M	60	-
Normal lung7	M	72	-
Normal lung8	M	72	-
Normal lung9	M	61	-
Normal lung10	M	70	-
Normal lung11	F	69	-
Normal lung12	M	70	-
Normal lung13	F	64	-
Normal lung14	F	72	-
Normal lung15	F	71	-

## 5 Conclusion

The identification of DE miRNAs holds promising potential for improving the diagnosis and treatment of various cancers. In our study, we identified a panel of ten miRNAs (miR-200c-3p, miR-210-3p, miR-10a-5p, miR-130b-3p, miR-146b-5p, miR-503-5p, miR-196b-5p, and miR-3934-5p) using NGS and RT-qPCR methods. This panel was able to distinguish LUAD-BM from normal brain tissue samples with excellent sensitivity and specificity in the Hungarian population. Differential expression analysis of 20 LUAD and 15 normal lung samples (selected from the TCGA database representing European populations), together with LUAD-BM samples, confirmed the dysregulation of six of the ten validated miRNAs. Among these, miR-200c-3p, miR-146b-5p, and miR-3934-5p differed significantly between primary LUAD and LUAD-BM, while miR-10a-5p, miR-210-3p, and miR-130b-3p showed progressive dysregulation along the normal lung–LUAD–LU-AD-BM axis. This study is the first to integrate brain tissue samples in the analysis of miRNA expression in LUAD-BM while simultaneously comparing LUAD-BM with nor-mal brain, primary LUAD, and normal lung tissues. Our findings are consistent with previously published data and support the crucial role of these miRNAs in the development of BM.

A limitation of this study is the relatively small sample size, highlighting the need for validation in larger independent cohorts. In addition, the lack of primary lung tumor samples restricted direct comparison between primary LUAD and LUAD brain metastases, which should be addressed in future studies using matched tumor pairs.

## Data Availability

The datasets analysed for this study can be found in the NCBI Gene Expression Omnibus (GEO) database at https://www.ncbi.nlm.nih.gov/geo/ and can be accessed with the accession number GSE284777 and GSE244332.
