# Supplementary material for: The role of miRNAs in the development of brain metastases originating from lung adenocarcinoma
Source: Front Genet. 2026 Feb 20;17:1769972. doi: 10.3389/fgene.2026.1769972 (PMC12962924; doi:10.3389/fgene.2026.1769972)
Supplement: Supplementary file 1 [file Table1.docx]

Supplementary Material

# Supplementary Tables

Table S1. List of the 118 significantly up-regulated and 111 significantly down-regulated miRNAs in tissue samples of LUAD-BM compared with their expression in Control group, listed according to their expression levels.

| BM-LUAD – Control (UP) | | | |
| --- | --- | --- | --- |
| Regulation | **miRNA** | **logFC** | **adj-P-Val** |
| UP | HSA-MIR-200C-3P | 9.236660023 | 3.65E-06 |
| UP | HSA-MIR-375-3P | 8.917612387 | 0.000556309 |
| UP | HSA-MIR-21-3P | 7.880115707 | 8.81E-05 |
| UP | HSA-MIR-200A-5P | 7.64026087 | 2.92E-05 |
| UP | HSA-MIR-210-3P | 7.372334767 | 8.81E-05 |
| UP | HSA-MIR-141-3P | 7.266670477 | 1.53E-06 |
| UP | HSA-MIR-200A-3P | 7.262304313 | 4.99E-05 |
| UP | HSA-MIR-10A-5P | 7.14916651 | 0.000864793 |
| UP | HSA-MIR-141-5P | 6.988811017 | 1.53E-06 |
| UP | HSA-MIR-200B-3P | 6.462992733 | 0.000176013 |
| UP | HSA-MIR-429 | 6.11923412 | 0.000136921 |
| UP | HSA-MIR-214-3P | 5.471112253 | 0.002182505 |
| UP | HSA-MIR-182-5P | 5.456388003 | 0.00114866 |
| UP | HSA-MIR-96-5P | 5.363271117 | 0.002222947 |
| UP | HSA-MIR-452-5P | 5.180669915 | 0.008812729 |
| UP | HSA-MIR-147B-3P | 4.991828427 | 0.002134568 |
| UP | HSA-MIR-21-5P | 4.799467967 | 0.00102287 |
| UP | HSA-MIR-148A-5P | 4.640099333 | 0.006581381 |
| UP | HSA-MIR-27A-5P | 4.59136857 | 0.000864793 |
| UP | HSA-MIR-130B-3P | 4.56850095 | 0.000694819 |
| UP | HSA-MIR-200B-5P | 4.552542093 | 0.000609721 |
| UP | HSA-MIR-576-3P | 4.512213725 | 0.000864793 |
| UP | HSA-MIR-214-5P | 4.457307649 | 0.009143627 |
| UP | HSA-MIR-1266-5P | 4.317679777 | 4.99E-05 |
| UP | HSA-MIR-183-5P | 4.274650367 | 0.003014693 |
| UP | HSA-MIR-19B-3P | 4.27427616 | 0.000493402 |
| UP | HSA-MIR-17-3P | 4.236468177 | 0.005510772 |
| UP | HSA-MIR-200C-5P | 4.158788717 | 0.00115944 |
| UP | HSA-MIR-146B-5P | 4.141913633 | 0.003331234 |
| UP | HSA-MIR-130A-5P | 4.00685542 | 0.015805705 |
| UP | HSA-MIR-210-5P | 3.892706127 | 0.005255699 |
| UP | HSA-MIR-503-5P | 3.881168772 | 0.000694819 |
| UP | HSA-MIR-10B-5P | 3.84279863 | 0.005056548 |
| UP | HSA-MIR-199B-5P | 3.823788847 | 0.001937291 |
| UP | HSA-MIR-146B-3P | 3.69141957 | 0.007730533 |
| UP | HSA-MIR-671-5P | 3.60600298 | 0.030288257 |
| UP | HSA-MIR-199A-5P | 3.600735727 | 0.006581381 |
| UP | HSA-MIR-196B-5P | 3.537931967 | 0.008812729 |
| UP | HSA-MIR-18A-3P | 3.484697967 | 0.004358794 |
| UP | HSA-MIR-130A-3P | 3.481217603 | 0.024714774 |
| UP | HSA-MIR-193A-3P | 3.329298732 | 0.008999351 |
| UP | HSA-MIR-146A-5P | 3.327944033 | 0.008409772 |
| UP | HSA-MIR-28-3P | 3.3240413 | 0.000704754 |
| UP | HSA-MIR-217-5P | 3.294969031 | 0.018569275 |
| UP | HSA-LET-7G-3P | 3.268452423 | 0.009143627 |
| UP | HSA-MIR-29A-5P | 3.196190774 | 0.01056158 |
| UP | HSA-MIR-25-5P | 3.076384117 | 0.001641439 |
| UP | HSA-MIR-152-5P | 3.052566855 | 0.001641439 |
| UP | HSA-MIR-6510-3P | 3.046231517 | 0.038542302 |
| UP | HSA-MIR-19A-3P | 3.045505593 | 0.002540307 |
| UP | HSA-MIR-34A-5P | 2.991419287 | 0.000556309 |
| UP | HSA-MIR-629-5P | 2.990596607 | 0.004329693 |
| UP | HSA-MIR-92A-1-5P | 2.94027276 | 0.01251271 |
| UP | HSA-MIR-7974 | 2.873277505 | 0.019601199 |
| UP | HSA-MIR-3934-5P | 2.835756267 | 0.001719874 |
| UP | HSA-MIR-551A | 2.828831933 | 0.029150583 |
| UP | HSA-MIR-425-5P | 2.759169697 | 0.008812729 |
| UP | HSA-MIR-365A-5P | 2.74598309 | 0.00953049 |
| UP | HSA-MIR-16-1-3P | 2.730569917 | 0.00131795 |
| UP | HSA-MIR-532-3P | 2.705852778 | 0.036670601 |
| UP | HSA-MIR-155-5P | 2.671169697 | 0.014949873 |
| UP | HSA-MIR-222-3P | 2.661845403 | 0.036716487 |
| UP | HSA-MIR-27A-3P | 2.641385457 | 0.000175742 |
| UP | HSA-MIR-29B-3P | 2.6203379 | 0.02411381 |
| UP | HSA-MIR-92B-5P | 2.611345245 | 0.030255635 |
| UP | HSA-MIR-148A-3P | 2.588536267 | 0.048887691 |
| UP | HSA-MIR-193A-5P | 2.52171865 | 0.00911739 |
| UP | HSA-MIR-193B-3P | 2.505837243 | 0.009143627 |
| UP | HSA-MIR-574-3P | 2.49276584 | 0.003406148 |
| UP | HSA-MIR-19B-1-5P | 2.46635921 | 0.010522418 |
| UP | HSA-MIR-106B-5P | 2.441687517 | 0.005540324 |
| UP | HSA-MIR-378A-3P | 2.411961633 | 0.008812729 |
| UP | HSA-MIR-1307-5P | 2.39173269 | 0.000442605 |
| UP | HSA-MIR-542-3P | 2.391408233 | 0.045669513 |
| UP | HSA-MIR-1304-3P | 2.368232861 | 0.000210294 |
| UP | HSA-MIR-23A-3P | 2.35986828 | 0.00114866 |
| UP | HSA-MIR-339-5P | 2.346736783 | 0.009764519 |
| UP | HSA-MIR-3158-3P | 2.326236223 | 0.025808226 |
| UP | HSA-MIR-362-3P | 2.32590141 | 0.038020371 |
| UP | HSA-MIR-493-3P | 2.324577893 | 0.027912004 |
| UP | HSA-MIR-378A-5P | 2.264352547 | 0.011221565 |
| UP | HSA-MIR-22-3P | 2.262929533 | 0.008999351 |
| UP | HSA-MIR-580-3P | 2.232460409 | 8.14E-05 |
| UP | HSA-MIR-505-3P | 2.231182017 | 0.039781135 |
| UP | HSA-MIR-4677-3P | 2.191528167 | 0.00359363 |
| UP | HSA-MIR-376B-3P | 2.149775003 | 0.049505881 |
| UP | HSA-MIR-186-5P | 2.131227967 | 0.000610139 |
| UP | HSA-MIR-1307-3P | 2.114028317 | 0.002246597 |
| UP | HSA-MIR-337-5P | 2.108369983 | 0.000864793 |
| UP | HSA-MIR-424-5P | 2.107859633 | 0.034224828 |
| UP | HSA-MIR-10399-5P | 2.067253733 | 0.013657696 |
| UP | HSA-MIR-4326 | 2.04759606 | 0.042655829 |
| UP | HSA-LET-7I-3P | 2.032722627 | 0.00545666 |
| UP | HSA-MIR-142-3P | 2.03172625 | 0.030288257 |
| UP | HSA-MIR-548E-3P | 1.9813464 | 0.002036376 |
| UP | HSA-MIR-93-3P | 1.960667077 | 0.00911739 |
| UP | HSA-MIR-125A-3P | 1.957534198 | 0.015186242 |
| UP | HSA-MIR-378I | 1.913434314 | 0.032070481 |
| UP | HSA-MIR-26B-3P | 1.828567633 | 0.000525746 |
| UP | HSA-MIR-3913-5P | 1.788795825 | 0.017680604 |
| UP | HSA-MIR-574-5P | 1.76133466 | 0.038542302 |
| UP | HSA-MIR-24-3P | 1.73405011 | 0.000981414 |
| UP | HSA-MIR-324-5P | 1.71783737 | 0.033224973 |
| UP | HSA-MIR-652-3P | 1.672133967 | 0.027793559 |
| UP | HSA-MIR-223-3P | 1.658850213 | 0.02411381 |
| UP | HSA-MIR-3679-5P | 1.59510198 | 0.00115944 |
| UP | HSA-MIR-25-3P | 1.570215133 | 0.008949067 |
| UP | HSA-MIR-422A | 1.554836955 | 0.027403134 |
| UP | HSA-MIR-34A-3P | 1.51915549 | 0.046982595 |
| UP | HSA-MIR-151A-3P | 1.503980433 | 0.033100541 |
| UP | HSA-MIR-151A-5P | 1.47987577 | 0.02411381 |
| UP | HSA-MIR-4470 | 1.450520083 | 0.011949205 |
| UP | HSA-MIR-152-3P | 1.4335093 | 0.006874491 |
| UP | HSA-MIR-423-5P | 1.329758567 | 0.007730533 |
| UP | HSA-MIR-7705 | 1.318997415 | 0.025808226 |
| UP | HSA-MIR-627-5P | 1.29050025 | 0.038316213 |
| UP | HSA-MIR-24-2-5P | 1.226608622 | 0.045669513 |
| UP | HSA-MIR-345-5P | 1.032724007 | 0.032430766 |

| BM-LUAD – Control (DOWN) | | | |
| --- | --- | --- | --- |
| Regulation | **miRNA** | **logFC** | **adj-P-Val** |
| DOWN | HSA-MIR-135A-5P | -5.677099155 | 0.00102287 |
| DOWN | HSA-MIR-323A-3P | -5.57043264 | 0.002134568 |
| DOWN | HSA-MIR-656-3P | -5.459175432 | 0.000704754 |
| DOWN | HSA-MIR-433-3P | -5.40984825 | 0.00131795 |
| DOWN | HSA-MIR-383-5P | -5.3643089 | 0.00102287 |
| DOWN | HSA-MIR-129-5P | -5.356577717 | 0.004358794 |
| DOWN | HSA-MIR-129-1-3P | -5.300697463 | 0.000864793 |
| DOWN | HSA-MIR-9-3P | -5.200237133 | 0.00142972 |
| DOWN | HSA-MIR-411-5P | -5.118635333 | 0.001445604 |
| DOWN | HSA-MIR-889-3P | -5.1087528 | 0.005035464 |
| DOWN | HSA-MIR-539-3P | -5.07898869 | 0.002134568 |
| DOWN | HSA-MIR-124-3P | -4.993781955 | 0.00102287 |
| DOWN | HSA-MIR-490-3P | -4.97197547 | 0.007673945 |
| DOWN | HSA-MIR-129-2-3P | -4.925791283 | 0.005035464 |
| DOWN | HSA-MIR-885-5P | -4.87559715 | 0.000556309 |
| DOWN | HSA-MIR-128-3P | -4.78856755 | 0.000400634 |
| DOWN | HSA-MIR-410-3P | -4.73009819 | 0.001038116 |
| DOWN | HSA-MIR-9-5P | -4.641059417 | 0.000694819 |
| DOWN | HSA-MIR-153-3P | -4.63807718 | 0.002540307 |
| DOWN | HSA-MIR-1197 | -4.59204851 | 0.003163322 |
| DOWN | HSA-MIR-139-5P | -4.528608833 | 0.002540307 |
| DOWN | HSA-MIR-124-5P | -4.495739055 | 0.000842647 |
| DOWN | HSA-MIR-543 | -4.264113293 | 0.003024773 |
| DOWN | HSA-MIR-628-5P | -4.250571483 | 3.49E-05 |
| DOWN | HSA-MIR-144-5P | -4.184598127 | 0.01733055 |
| DOWN | HSA-MIR-598-3P | -4.116230073 | 0.00142972 |
| DOWN | HSA-MIR-329-3P | -4.092149117 | 0.000864793 |
| DOWN | HSA-MIR-204-5P | -4.032331383 | 0.005476797 |
| DOWN | HSA-MIR-219A-2-3P | -4.026441783 | 0.027552583 |
| DOWN | HSA-MIR-499A-5P | -3.923567333 | 0.002747236 |
| DOWN | HSA-MIR-369-3P | -3.808176873 | 0.001719874 |
| DOWN | HSA-MIR-485-3P | -3.772271413 | 0.001641439 |
| DOWN | HSA-MIR-218-5P | -3.72422015 | 0.00142972 |
| DOWN | HSA-MIR-95-3P | -3.640190923 | 0.010218107 |
| DOWN | HSA-MIR-139-3P | -3.638678467 | 0.022096004 |
| DOWN | HSA-MIR-340-3P | -3.63410946 | 0.001395812 |
| DOWN | HSA-MIR-495-3P | -3.6263224 | 0.000441252 |
| DOWN | HSA-MIR-1-3P | -3.61337572 | 0.00318162 |
| DOWN | HSA-MIR-592 | -3.574869177 | 0.005201727 |
| DOWN | HSA-MIR-487B-3P | -3.537598083 | 0.00142972 |
| DOWN | HSA-MIR-409-5P | -3.53189585 | 0.006600615 |
| DOWN | HSA-MIR-539-5P | -3.481070982 | 0.00142972 |
| DOWN | HSA-MIR-655-3P | -3.471859407 | 0.001479195 |
| DOWN | HSA-MIR-204-3P | -3.39126573 | 0.00142972 |
| DOWN | HSA-MIR-1224-3P | -3.310660307 | 0.008812729 |
| DOWN | HSA-MIR-330-5P | -3.264481797 | 0.007730533 |
| DOWN | HSA-MIR-340-5P | -3.2176065 | 0.001719874 |
| DOWN | HSA-MIR-487A-3P | -3.180631437 | 0.00820955 |
| DOWN | HSA-MIR-370-3P | -3.11383495 | 0.006087571 |
| DOWN | HSA-MIR-4705 | -3.05259725 | 0.002540307 |
| DOWN | HSA-MIR-154-3P | -2.968265933 | 0.002036376 |
| DOWN | HSA-MIR-346 | -2.939926153 | 0.022003077 |
| DOWN | HSA-MIR-758-3P | -2.909672317 | 0.000400634 |
| DOWN | HSA-MIR-382-3P | -2.900627573 | 0.00115944 |
| DOWN | HSA-MIR-125B-2-3P | -2.872820563 | 0.006874491 |
| DOWN | HSA-MIR-1185-1-3P | -2.868153963 | 0.016317227 |
| DOWN | HSA-MIR-374B-3P | -2.864030063 | 0.016674744 |
| DOWN | HSA-MIR-132-5P | -2.82305503 | 0.050716259 |
| DOWN | HSA-MIR-412-5P | -2.807369965 | 0.016108014 |
| DOWN | HSA-MIR-3912-3P | -2.800203345 | 0.000685291 |
| DOWN | HSA-MIR-301A-5P | -2.787718405 | 0.005225632 |
| DOWN | HSA-MIR-136-3P | -2.777393317 | 0.005169651 |
| DOWN | HSA-MIR-421 | -2.768843487 | 0.006600615 |
| DOWN | HSA-MIR-431-3P | -2.754329322 | 0.005346272 |
| DOWN | HSA-MIR-6868-3P | -2.744870797 | 0.018913765 |
| DOWN | HSA-MIR-363-3P | -2.737095683 | 0.009764519 |
| DOWN | HSA-MIR-379-5P | -2.697472933 | 0.000864793 |
| DOWN | HSA-MIR-767-5P | -2.663371893 | 0.010696201 |
| DOWN | HSA-MIR-149-5P | -2.615756877 | 0.015186242 |
| DOWN | HSA-MIR-1296-5P | -2.555100673 | 0.000864793 |
| DOWN | HSA-MIR-3085-3P | -2.41586685 | 0.010038674 |
| DOWN | HSA-MIR-3200-3P | -2.36182364 | 0.036716487 |
| DOWN | HSA-MIR-1249-3P | -2.295990405 | 0.035599537 |
| DOWN | HSA-MIR-381-3P | -2.290371667 | 0.032070481 |
| DOWN | HSA-MIR-7-5P | -2.288056767 | 0.011949205 |
| DOWN | HSA-MIR-105-5P | -2.27776187 | 0.002747236 |
| DOWN | HSA-MIR-195-5P | -2.255198033 | 0.031485116 |
| DOWN | HSA-MIR-487A-5P | -2.239112463 | 0.012224073 |
| DOWN | HSA-MIR-20B-5P | -2.222400673 | 0.033342795 |
| DOWN | HSA-MIR-299-5P | -2.189116 | 0.035204957 |
| DOWN | HSA-MIR-330-3P | -2.168574693 | 0.006087571 |
| DOWN | HSA-MIR-138-2-3P | -2.098848948 | 0.009143627 |
| DOWN | HSA-LET-7E-3P | -2.04441845 | 0.046590319 |
| DOWN | HSA-MIR-382-5P | -2.038298283 | 0.010730818 |
| DOWN | HSA-MIR-885-3P | -2.005152677 | 0.010897065 |
| DOWN | HSA-MIR-107 | -1.988707633 | 0.009143627 |
| DOWN | HSA-MIR-328-3P | -1.937035017 | 0.008949067 |
| DOWN | HSA-MIR-140-5P | -1.92526321 | 0.003046042 |
| DOWN | HSA-MIR-3943 | -1.917662357 | 0.02411381 |
| DOWN | HSA-MIR-433-5P | -1.854953892 | 0.00114866 |
| DOWN | HSA-MIR-6516-3P | -1.831326898 | 0.039748838 |
| DOWN | HSA-MIR-1271-5P | -1.785292953 | 0.013657696 |
| DOWN | HSA-MIR-3117-3P | -1.689891913 | 0.040568802 |
| DOWN | HSA-MIR-1185-5P | -1.684777007 | 0.042655829 |
| DOWN | HSA-MIR-181D-5P | -1.661703017 | 0.006855697 |
| DOWN | HSA-MIR-369-5P | -1.607546086 | 0.018320537 |
| DOWN | HSA-MIR-30C-5P | -1.605568467 | 0.01259573 |
| DOWN | HSA-MIR-154-5P | -1.602639447 | 0.014089268 |
| DOWN | HSA-MIR-125B-5P | -1.5959694 | 0.006874491 |
| DOWN | HSA-MIR-708-5P | -1.58323079 | 0.037534323 |
| DOWN | HSA-MIR-1185-2-3P | -1.565525928 | 0.031697013 |
| DOWN | HSA-MIR-26A-5P | -1.511784633 | 0.007730533 |
| DOWN | HSA-MIR-494-3P | -1.500415877 | 0.008227351 |
| DOWN | HSA-MIR-488-5P | -1.44039635 | 0.015805705 |
| DOWN | HSA-LET-7E-5P | -1.3966183 | 0.045669513 |
| DOWN | HSA-LET-7D-3P | -1.391808933 | 0.00114866 |
| DOWN | HSA-MIR-1179 | -1.391284207 | 0.003331234 |
| DOWN | HSA-LET-7A-5P | -1.37193175 | 0.030593293 |
| DOWN | HSA-MIR-6866-5P | -1.359483232 | 0.01323489 |
| DOWN | HSA-LET-7G-5P | -1.2223313 | 0.035599537 |
| DOWN | HSA-MIR-744-5P | -1.054788577 | 0.027552583 |
